# Circulation dynamics of West Nile virus in Germany, 2023 and 2024

**DOI:** 10.1186/s12985-025-03043-8

**Published:** 2025-12-18

**Authors:** Anne Schwarzer, Franziska Schopf, Martin H. Groschup, Sabine Bock, Kristin Heenemann, Louise Herms, Anke Himmelreich, Susanne Kenklies, Jochen Kilwinski, Andrea Konrath, Friederike Michel, Kerstin Müller, Grit Priemer, Ronja Rahner, Claudia Sauerwald, Nelly Scuda, Timo Siempelkamp, Jasmin Skuballa, Hubert Wonnemann, Markus Keller, Thomas W. Vahlenkamp, Ute Ziegler, Balal Sadeghi

**Affiliations:** 1 Federal Research Institute for Animal Health, Institute of Novel and Emerging Infectious Diseases, Friedrich-Loeffler-Institut, Südufer 10, 17493 Greifswald-Insel Riems, Germany; 2 German Center of Infection Research (DZIF), Partner site Hamburg- Lübeck-Borstel-Riems, Südufer 10, 17493 Greifswald-Insel Riems, Germany; 3 Berlin-Brandenburg State Laboratory, Gerhard-Neumann-Straße 2, 15236 Frankfurt (Oder), Germany; 4 Institute of Virology, Center for Infectious Diseases, Faculty of Veterinary Medicine, Leipzig University, An den Tierkliniken 29, 04103 Leipzig, Germany; 5 Lower Saxony State Office for Consumer Protection and Food Safety, Eintrachtweg 17, 30173 Hannover, Germany; 6 Institute for Hygiene and Environment, Marckmannstraße 129A, 20539 Hamburg, Germany; 7 State Office for Consumer Protection Saxony-Anhalt, Haferbreiter Weg 132-135, 39576 Stendal, Germany; 8 Chemical and Veterinary Investigation Office Westfalen, Zur Taubeneiche 10-12, 59821 Arnsberg, Germany; 9 Saxon State Laboratory of Health and Veterinary Affairs, Bahnhofstraße 58-60, 04158 Leipzig, Germany; 10 Saxon State Laboratory of Health and Veterinary Affairs, Jägerstraße 8/10, 01099 Dresden, Germany; 11 Division for Small Mammals, Birds and Reptiles, School of Veterinary Medicine, Small Animal Clinic, Freie Universität Berlin, Oertzenweg 19B, 14163 Berlin, Germany; 12 State Office of Agriculture, Food Safety and Fisheries Mecklenburg- Western Pomerania, Thierfelderstraße 18, 18059 Rostock, Germany; 13 Clinic for Birds, Reptiles, Amphibians and Fish, Justus Liebig University, Frankfurter Straße 114, 35392 Gießen, Germany; 14 Department of Veterinary Medicine, Hessian State Laboratory, Schubertstraße 60, 35392 Gießen, Germany; 15 Bavarian Health and Food Safety Authority, Eggenreuther Weg 43, 91058 Erlangen, Germany; 16 Thuringian State Office for Consumer Protection, Tennstedter Straße 8/9, 99947 Bad Langensalza, Germany; 17 State Institute for Chemical and Veterinary Analysis Karlsruhe, Weißenburger Straße 3, 76187 Karlsruhe, Germany; 18 State Laboratory of Schleswig-Holstein, Max-Eyth-Straße 5, 24537 Neumünster, Germany

**Keywords:** West Nile virus, Zoonosis, Monitoring, Flavivirus, Phylogeny, Germany

## Abstract

**Background:**

West Nile virus (WNV) is spreading rapidly to numerous countries in Europe. WNV maintains an enzootic cycle with many bird species as key amplifying hosts and mosquitoes of the *Culex pipiens* complex as main vectors. Worldwide, nine WNV lineages have been described so far. The objective of this study was to elucidate the genetic diversity of WNV in Germany in 2023 and 2024 with a focus on birds as reservoir hosts.

**Methods:**

A total of 86 samples (25 from 2023, 61 from 2024) from dead and live birds submitted by German state veterinary laboratories and as part of a nationwide wild bird surveillance network. Samples were selected for whole genome sequencing (WGS) based on bird species, Ct value, and location. Viral RNA was extracted from bird samples and submitted to two WNV specific reverse transcription quantitative real-time polymerase chain reaction protocols. Subsequently, viral RNAs were amplified, WGS was performed using MinION technology and WGS data were processed to determine lineages and clusters.

**Results:**

The majority of WNV RNA positive birds selected for WGS in 2023 and 2024 were Eurasian Goshawks (*Astur gentilis*, *n* = 45). In 2023, WNV RNA positive birds were identified in only six federal states, as opposed to 13 federal states in 2024. All WNV RNA positive bird samples from 2023 to 2024 belonged to WNV Lineage 2 (WNV-2). Of the 86 sequenced samples, ~ 73% clustered within subcluster 2.5.3.4.3c. This subcluster, which also includes closely related sequences from Austria, Czech Republic and Slovakia, represents the dominant circulating variant. The remaining sequences (~ 27%) grouped within cluster 2.5.3.2, demonstrating the co-circulation of at least two genetically distinct WNV-2 variants in Germany.

**Conclusions:**

These findings highlight the ongoing geographic expansion and local establishment of WNV-2 seven years after the initial detection of this virus in Germany, characterized by continuous virus evolution and the predominance of subcluster 2.5.3.4.3c, which suggests regional connectivity of transmission chains, underscoring the importance of transboundary surveillance and coordinated control measures.

**Supplementary Information:**

The online version contains supplementary material available at 10.1186/s12985-025-03043-8.

## Background

Mosquito-borne arboviruses, such as West Nile virus (WNV, *Orthoflavivirus nilense*), are emerging pathogens worldwide. Their spread is caused by climate induced changes, especially in temperate regions where vector-host interactions are increasingly modulated by environmental transformations [1].

WNV was first isolated in the West Nile District of Uganda. In Europe, WNV has emerged with outbreaks first detected in the late 20th century [1]. So far, nine WNV lineages have been described, of which lineage 1 (WNV-1) and lineage 2 (WNV-2) are associated with human disease [2]. While WNV-1 has been identified in North America, North Africa, Europe and Australia, WNV-2 has its origins in Southern Africa and is now also present in central and eastern Europe [1]. WNV has spread across various European countries, such as Italy, Greece, Romania, France and Hungary [3]. In 2018, Europe experienced its most substantial WNV outbreak season, affecting 11 countries and leading to 1,548 mosquito-borne infections in humans [4]. Human infections can be asymptomatic or cause only mild flu-like symptoms, but can also lead to severe neurological symptoms with fatal outcomes [5]. In Germany, however, it was dead birds in which the first cases of WNV were detected in 2018 [6]. WNV is primarily maintained in an enzootic cycle between avian hosts and mosquitoes of the *Culex pipiens* complex, with different bird species acting as key amplifying hosts [7, 8].

Global climate change, particularly warming trends, is expected to have a significant impact on temperate regions. This will affect the reproductive patterns of vectors and shorten the extrinsic arboviral incubation period in mosquitoes, thereby disrupting the ecological balance, resulting in an escalation of arbovirus prevalence in previously low-risk areas [9, 10]. The WNV epidemic in Germany in 2018 and 2019 can be attributed to elevated regional temperatures, which favored the establishment and rapid spread of the virus and influenced its dynamics in both mosquitoes and avian hosts [11, 12].

**Fig. 1:**
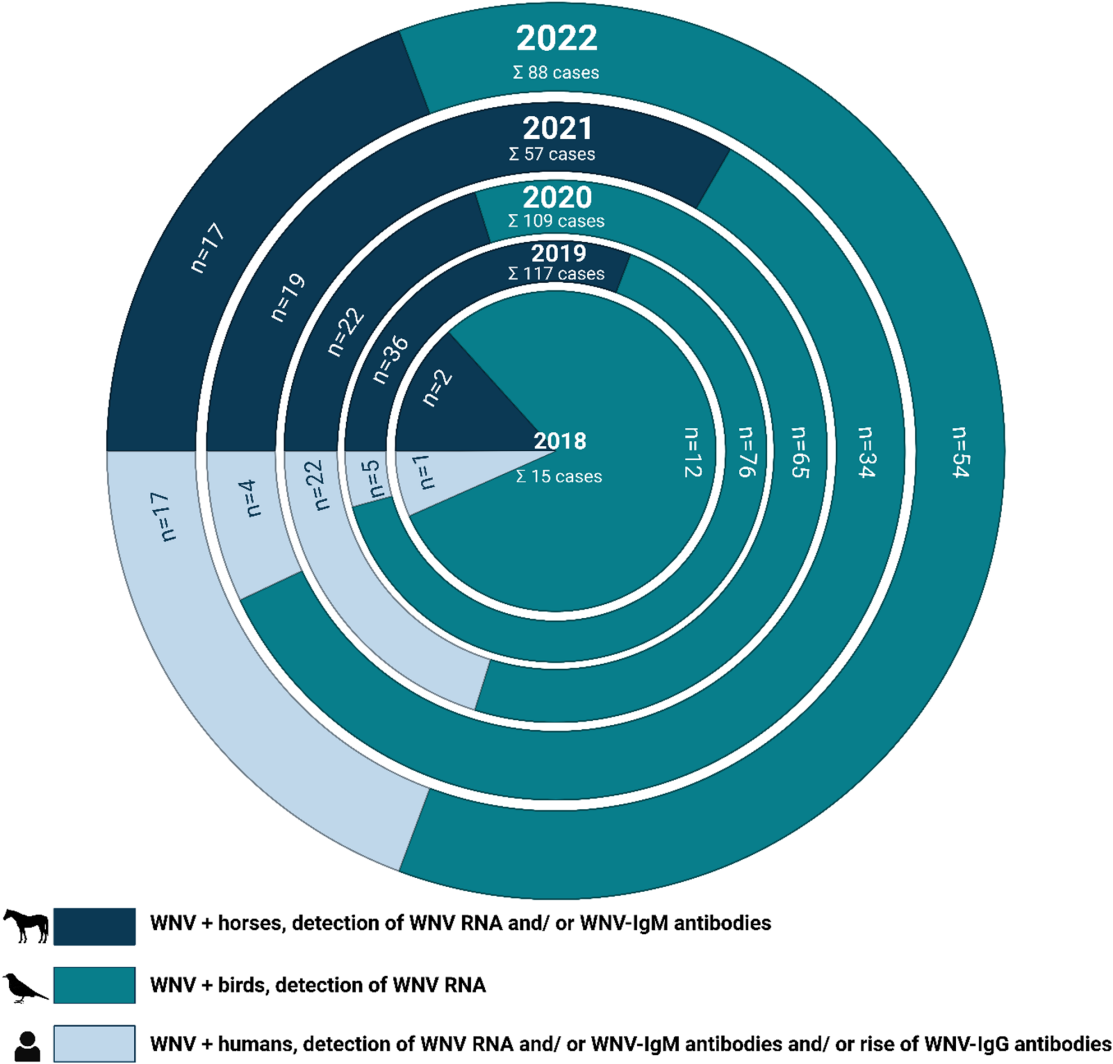
Number of detected autochthonous cases of West Nile virus infection in Germany from 2018 to 2022. The numbers of cases in horses, birds, and humans are shown in different shades of blue for each year

To provide a comprehensive overview, Fig. 1 illustrates the autochthonous WNV cases in Germany in humans, horses, and birds in the years from the first WNV detection in Germany 2018 to 2022 [13]. Notifiable cases in horses (detection of WNV RNA and/or WNV-IgM antibodies), birds (detection of WNV RNA) and humans (detection of WNV RNA and/or WNV-IgM antibodies and/or rise of WNV-IgG antibodies after repeated testing) are shown. Only human cases that have been reported in accordance with the German “Protection against infection law” are included in the figure. Eight additional human cases from the year 2020, which were reported in accordance with the German “Transfusion law”, are not shown [14]. It is also noteworthy that although the sole human case in 2018 was documented as an autochthonous case, it was not an infection transmitted by a mosquito bite. Rather, it was an infection that occurred during the dissection of a WNV positive bird [14]. The data on human cases were contributed by the Robert Koch Institute (RKI) [15]. The cases in horses and birds (according to the German “Regulation on notifiable animal diseases”) were confirmed by the National Reference Laboratory (NRL) for WNV at the Friedrich-Loeffler-Institut (FLI) [11, 16–23].

This study investigates the circulation and genetic diversity of WNV in Germany, with particular emphasis on the years 2023 and 2024. Using whole genome sequencing (WGS), it explores the evolutionary dynamics of the virus, its persistence in wild and zoo birds as amplifier and reservoir hosts and its transmission through mosquitoes as vectors. The high-resolution workflow defined by Santos et al. [24] was used to accurately trace the enzootic maintenance and local evolution of WNV in Germany. The results will contribute to a better understanding at both, European and global scales and provide insights to design better public health strategies and vector control measures.

## Methods

### Sample collection

Most of the samples used in this study were submitted to the NRL for WNV at the FLI for confirmation of WNV infection. Additionally, some samples were collected within a nationwide wild bird surveillance network as described by Schopf et al. [23]. Tissue and/or blood samples from 86 WNV positive birds from different species that were found sick or died in 2023 (25 samples) and 2024 (61 samples) were included in the present study. Detailed information on the samples can be found in Tables 1 and 2. More detailed information on sample origin and the material used for WGS can be found in the Supplementary Tables S1 and S2 for 2023 and 2024, respectively. In cases where multiple organs were available as sample material, the material with the lowest Ct value in the reverse transcription quantitative real-time polymerase chain reaction (RT-qPCR) was selected for further WGS analysis. Occasionally, RNA supplied by the contributors was used.

**Table 1:** Overview of WNV RT-qPCR positive bird samples in 2023

Bird order	Bird species (common name)	Bird species (scientific name)	Housing	Sample origin: federal states	Number of WNV RNA positive dead bird samples (NRL confirmed)
Accipitriformes	Eurasian Goshawk	*Astur gentilis*	wild	BB, BE, ST	14
Anseriformes	Brant Goose	*Branta bernicla*	zoo	BB	1
Charadriiformes	Common Redshank	*Tringa totanus*	zoo	SN	1
Falconiformes	European Kestrel	*Falco tinnunculus*	wild	BB, ST	1 (1)
Psittaciformes	Swift Parrot	*Lathamus discolor*	zoo	ST	1
Strigiformes	Snowy Owl	*Bubo scandiacus*	zoo	RP, SN, TH	3
	Tawny Owl	*Strix aluco*	zoo	ST	1 (1)
	Northern Hawk-Owl	*Surnia ulula*	zoo	SN	1
					**Subtotal: 25**
Additionally: blood samples from live birds from wild bird surveillance network					2
					**Total: 27**

Federal States are abbreviated as follows: BE = Berlin, BB = Brandenburg, RP = Rhineland-Palatinate, SN = Saxony, ST = Saxony-Anhalt, TH = Thuringia. Bird samples that were WNV RNA positive but were not whole genome sequenced are mentioned in brackets. Additional information on the samples can be found in supplementary table 1.

**Table 2:** Overview of WNV RT-qPCR positive bird samples in 2024

Bird order	Bird species (common name)	Bird species (scientific name)	Housing	Sample origin: federal states	Number of WNV RNA positive dead bird samples (NRL confirmed)
Accipitriformes	Eurasian Goshawk	*Astur gentilis*	wild, aviary	BB, BE, BW, HE, HH, NW, SH, TH	30 (1)
	Common Buzzard	*Buteo buteo*	wild	BE	1
	Bald Eagle	*Haliaeetus leucocephalus*	zoo	NI	1
Anseriformes	Indian Runner	*Anas platyrhynchos domesticus*	aviary	BB	(1)
Charadriiformes	Common Ringed Plover	*Charadrius hiaticula*	zoo	SN	1
	Black-tailed Gull	*Larus crassirostris*	zoo	BB	(1)
	Eurasian Curlew	*Numenius arquata*	zoo	BE	(1)
Falconiformes	American Kestrel	*Falco sparverius*	aviary	SN	(1)
	Saker Falcon	*Falco cherrug*	aviary	BB	1
Galliformes	Golden Pheasant	*Chrysolophus pictus*	zoo	SN	1
Passeriformes	Eurasian Blackbird	*Turdus merula*	wild	NI	1
	Blue Tit	*Cyanistes caeruleus*	wild	HE	1
	Great Tit	*Parus major*	wild	BY	1
	European Greenfinch	*Chloris chloris*	wild	ST	1
	Hawfinch	*Coccothraustes coccothraustes*	wild	BB	(1)
	Hooded Crow	*Corvus cornix*	wild	BE	1
	Domestic Canary	*Serinus canaria forma domestica*	aviary	ST	2
	Red-throated Parrotfinch	*Erythrura psittacea*	zoo	BE	(1)
	Red-winged Starling	*Onychognathus morio*	zoo	ST	1
	Unspecified sparrow	*Passer spp.*	wild	ST	1
Pelecaniformes	American White Pelican	*Pelecanus erythrorhynchos*	zoo	BB	(1)
Phoenicopteriformes	American Flamingo	*Phoenicopterus ruber*	zoo	BB	(2)
	Chilean Flamingo	*Phoenicopterus chilensis*	zoo	BE, SN	2 (1)
Psittaciformes	Coconut Lorikeet	*Trichoglossus haematodus*	zoo	ST	1
	Rainbow Lorikeet	*Trichoglossus moluccanus*	zoo	TH	1
	Scarlet-breasted Lorikeet	*Trichoglossus forsteni mitchellii*	zoo	BE	1 (4)
	Yellow-tailed Black Cockatoo	*Zanda funerea*	aviary	BB	(1)
Strigiformes	Boreal Owl	*Aegolius funereus*	zoo	SN	1
	Great Grey Owl	*Strix nebulosa*	zoo	MV	1 (1)
	Snowy Owl	*Bubo scandiacus*	zoo	MV, NW, SN, ST	5 (2)
	Tawny Owl	*Strix aluco*	wild, zoo	MV, NI	3
Tinamiformes	Chilean Tinamou	*Nothoprocta perdicaria*	zoo	ST	1 (3)
					**Subtotal: 82**
Additionally: blood samples from live birds from wild bird surveillance network					1
					**Total: 83**

Federal States are abbreviated as follows: BW = Baden-Wurttemberg, BY = Bavaria, BE = Berlin, BB = Brandenburg, HH = Hamburg, HE = Hesse, NI = Lower Saxony, MV = Mecklenburg-Western Pomerania, NW = North Rhine-Westphalia, SN = Saxony, ST = Saxony-Anhalt, SH = Schleswig-Holstein, TH = Thuringia. Bird samples that were WNV RNA positive but were not whole genome sequenced are mentioned in brackets. Additional information on the samples can be found in supplementary Table 2

### Molecular investigations

Viral RNA for RT-qPCR and WGS was extracted at the FLI from cruor or organ material using the RNeasy Mini Kit (Qiagen, Hilden, Germany) according to the manufacturer`s instructions. This RNA was tested in a RT-qPCR protocol for specific WNV detection (assay 1) [25], which uses WNV-specific 5` non-translation region primers and probe. To confirm the results, positive and inconclusive RNA samples were further screened by a RT-qPCR targeting the genomic NS2A region (assay 2) [25].

### Selection of bird samples for whole genome sequencing

In general, dead bird samples from the NRL were selected in such a way that if several bird samples were submitted in close proximity to each other, or even in the same aviary, only one sample was selected for WGS to increase time and cost efficiency. Furthermore, some samples from known WNV endemic areas were excluded, due to the substantial amount of WGS that has already been conducted in these regions [11, 24]. Prior to WGS, all WNV RNA positive samples with a Ct value >30 according to RT-qPCR results were excluded from the panel to ensure high quality results. Tables 1 and 2 provide information on the whole sample panel. Supplementary Tables S1 and S2 provide extensive information on the whole genome sequenced WNV positive bird samples from 2023 to 2024. Out of the 110 WNV RNA positive bird samples (107 dead birds from the NRL and three live birds of the wild bird monitoring) in 2023 and 2024, 86 samples were included in the phylogenetical analyses. For 2023 (Table 1), 23 out of the 25 WNV RT-qPCR positive samples confirmed by the NRL and two WNV RT-qPCR positive blood samples obtained from live birds of the wild bird monitoring (Table S1) were whole genome sequenced. These two blood samples (one Common Wood Pigeon (*Columba palumbus*) and one Eurasian Jay (*Garrulus glandarius*)) from the wild bird monitoring panel were selected because of the affected species. Three Eurasian Goshawks (*Astur gentilis)* as listed in Table S1 died during the course of infection. Blood cruor was available in the early stages of infection and whole genome sequenced, and their organs were tested later in the NRL using RT-qPCR, which is why they were first sampled as live birds and later as deceased birds. For 2024, tissue samples from 60 birds out of the dead bird panel were selected for WGS (Table 2). Additionally, one blood sample of a live Eurasian Goshawk from the wild bird monitoring was whole genome sequenced. This bird originated from the federal state of Hesse, whereof only a single bird was available for analysis at the NRL.

### Viral whole genome sequencing and phylogenetic analyses

To prepare the samples for WGS, the extracted RNA was reverse transcribed using non-specific primers. Afterwards, only the WNV derived cDNA was targeted for amplification by using two seperate oligonucleotide primer sets [26] creating overlapping amplicons in two sepearate reactions. The resulting amplicons were approximately 500 base pairs long [23]. For the subsequent processing of amplicons for WGS using MinION technology (Oxford Nanopore Technologies (ONT), Oxford Science Park, United Kingdom) the most recent kits for barcode ligation (Native Barcoding Kit 24 V14, REF: SQK-NBD114.24, ONT and NEBNext Quick Ligation Module, Blunt/TA Ligase Master Mix, M 0367 L, New England Biolabs), library preparation (Ligation Sequencing Kit XL, SQK-LSK1110-XL, ONT) and flow cell priming (Flow Cell Priming Kit, EXP-FLP002, ONT) as well as a new generation of flow cells (MinION GridION Flow Cell R10 Version, ONT) were utilized in comparison to previously described protocols [23].

Following WGS, Fast5 raw reads were basecalled with high accuracy, and demultiplexed using the Mk1C sequencer (Guppy v6.5.7, ONT). Subsequent processing, including additional demultiplexing, adapter removal, quality filtering, and consensus sequence assembly, was performed using the VirDetector pipeline [27]. This automated workflow is tailored for nanopore WGS data and facilitates rapid detection and characterization of viral genomes. Sequence alignment was conducted using the Clustal W algorithm within the MEGA v.11 software [28]. The most suitable nucleotide substitution model was selected with jModelTest v.2, using the Bayesian Information Criterion (BIC) for model selection. The best-fitting substitution model identified was GTR + gamma (G) [29]. Maximum likelihood (ML) phylogenetic trees were constructed using PAUP* v.4 [30], applying the subtree-pruning-regrafting branch-swapping algorithm for heuristic tree searches. Bootstrap analysis with 1000 replicates was performed to assess tree robustness, and the final trees were visualized using FigTree v1.4.3 [31] and iTOL [32].

For the classification of WNV lineages, the workflow described by Santos et al. was followed [24]. Full coding sequences were analyzed using the Affinity Propagation Clustering algorithm and Agglomerative Hierarchical Clustering, both implemented in the R package ‘apcluster’ v1.4.13 within RStudio (v2022.02.2–485). Finally, the trees generated and the lineage clusters inferred using the apcluster workflow were merged, and the combined results were visualized as one consolidated phylogenetic figure. For evolutionary dynamic analyses and to determine the time to the most recent common ancestor (tMRCA), the Bayesian Markov chain Monte Carlo (MCMC) method was performed using BEAST v2.6.6 package using a Coalescent Constant Size tree prior [33]. We performed two separate phylogenetic analyses based on different sequence datasets. The first analysis used sequences exclusively from Germany to focus on national lineage diversity. The second analysis incorporated sequences from multiple countries, including Austria, Hungary, Greece, Serbia, and others, to capture a broader geographic context and evolutionary relationships across the region. A detailed breakdown of the sequences, including their country of origin and total numbers, is provided in the Supplementary File F1. In these analyses, a GTR + G substitution model and a strict clock model were applied [34]. MCMC was set to 100,000,000 generations (sampling every 2,500 steps). Log files were analyzed in Tracer v1.7.1 to check effective sampling size values (>200 indicated sufficient sampling). The maximum clade credibility (MCC) tree was generated in the Tree Annotator v1.8.4, with a default burn-in of 10%. The MCC tree was visualized in the FigTree program v1.4.3 [31].

## Results

### RT-qPCR results

In 2023 and 2024, 25 and 82 dead bird samples were confirmed to be WNV RT-qPCR positive, respectively (Tables 1 and 2). Additionally, the dead bird panel was supplemented with positive samples from live wild birds as part of a nationwide surveillance network [23]. In 2023, 23 samples were selected for WGS from the dead bird panel, with the selection supplemented by two samples from the live wild bird panel. Three Eurasian Goshawks were included in both the wild bird panel and the dead bird panel (see Table S1). Blood samples of these three birds were initially sent to the FLI, followed by organ samples as the disease progressed. In 2024, 60 dead bird samples from the dead bird panel were whole genome sequenced, and one sample from the live wild bird panel was added. Consequently, a total of 86 samples (25 in 2023 and 61 in 2024) were subjected to WGS within the present study. The majority of the birds tested positive for WNV specific RNA in 2023 and 2024 were Eurasian Goshawks (*n* = 45). In 2023, Eurasian Goshawks accounted for 56% of WNV positive dead birds, and approximately 38% in 2024. Furthermore, a high number of WNV positive Snowy Owls (*Bubo scandiacus*) was observed, with three cases in 2023 and seven cases in 2024. These belonged to the order Strigiformes, of which a total of six (24% of the dead bird panel) and 13 (~ 16% of the dead bird panel) WNV positive birds were found in 2023 and 2024, respectively. In addition, bird species in Germany for which WNV has not yet been whole genome sequenced, or has been sequenced very rarely, were also included in the WGS panel. This included one Brant Goose (*Branta bernicla*, 2023), one Swift Parrot (*Lathamus discolor*, 2023), one Eurasian Jay (2023), one Common Wood Pigeon (2023) and one Common Ringed Plover (*Charadrius hiaticula*, 2024). In 2024, Lorikeets (*Trichoglossus sp.*, *n* = 7) were also frequently affected as zoo birds, and Chilean Tinamous (*Nothoprocta perdicaria*, *n* = 4) from a zoo in Saxony-Anhalt accounted for approximately 5% of the birds confirmed by the NRL.

In 2023, WNV RNA positive birds were found in six different federal states, whereas in 2024 the positive bird samples originated from 13 federal states.

All WNV RNA positive bird samples over both study years belonged to WNV-2. The start of the WNV season in 2023 is dated July 8, when a Eurasian Goshawk was found dead in Berlin and tested positive via WNV RT-qPCR. The last documented case of 2023 was a Eurasian Goshawk also from Berlin which was found dead on October 6. In 2024, the WNV season began with a positive Eurasian Goshawk from Berlin, which was found again as early as July 5, while the last case, also a Eurasian Goshawk, occurred as late as November 21 in southwestern Germany (Baden-Wurttemberg). Further information such as date of death and origin of all birds can be found in the Supplementary Tables S1 and S2.

### Phylogenetic analysis and subcluster distribution of WNV RNA positive birds

Phylogenetic analysis showed that all 86 sequenced WNV positive samples from 2023 to 2024 belonged to WNV-2, specifically the 2.5.3 subclade.

**Fig. 2:**
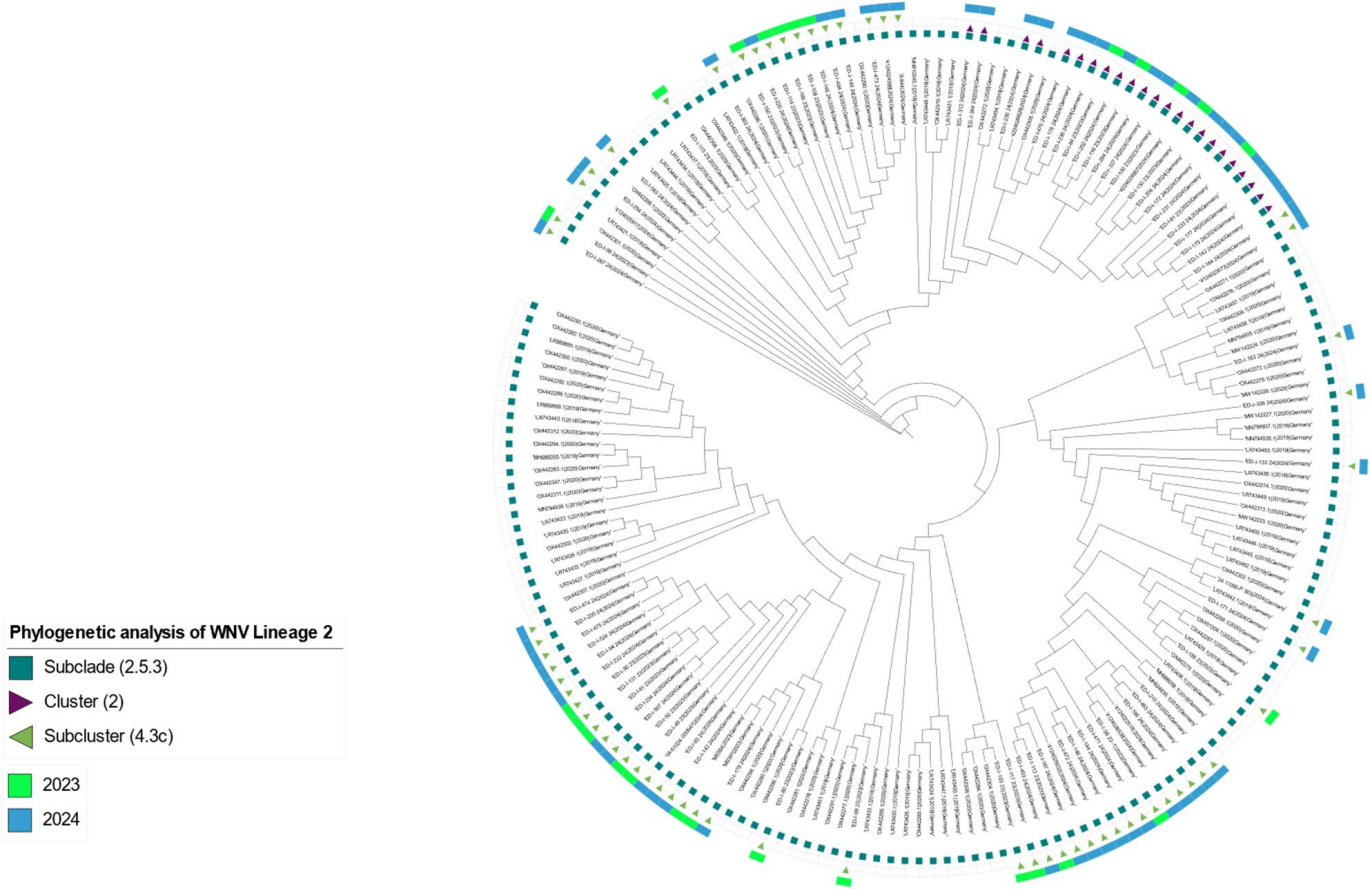
Phylogenetic tree of WNV sequences from Germany inferred by ML analysis. To classify WNV lineages objectively, clustering methods including Full Affinity Propagation Clustering and Agglomerative Hierarchical Clustering were applied to genetic distances derived from the ML phylogeny. Colored rectangles indicate year of sampling for new sequences acquired in this study (neon green = 2023, blue = 2024). The lineage classification results indicated that all WNV samples in Germany belong to Subclade 2.5.3 (petrol-colored squares). Triangles denote cluster or subcluster affiliation (purple = 2.5.3.2, green = 2.5.3.4.3c)

Among these, 63 sequences (~ 73%) grouped within subcluster 2.5.3.4.3c, indicating it as the dominant circulating variant. In 2023, 80.0% (20/25) of sequences belonged to this subcluster, compared to ~ 70.5% (43/61) in 2024. The remaining 23 sequences from both years (~ 27%) fell within a separate cluster 2.5.3.2 (Fig. 2).

**Fig. 3:**
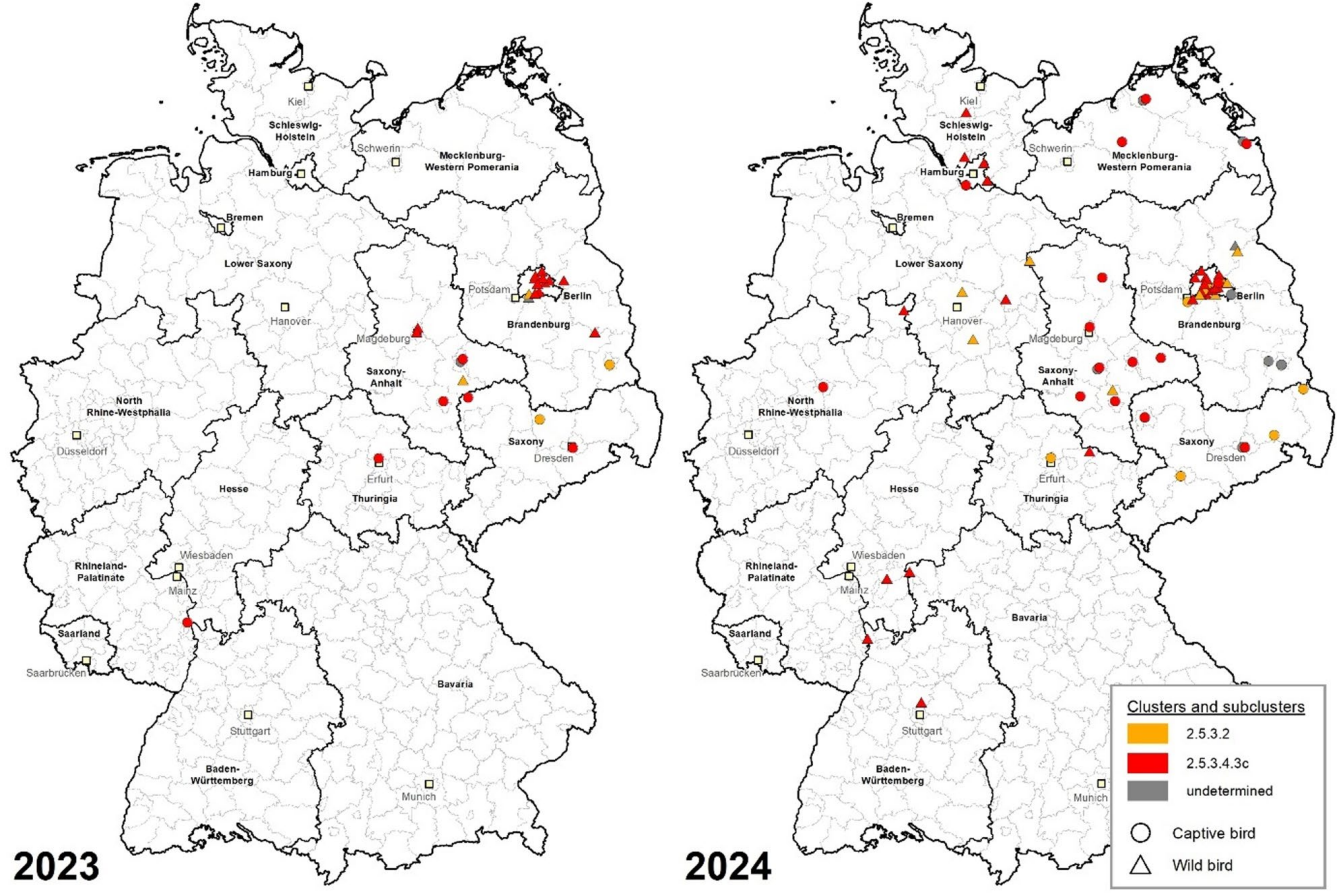
Geographical origin of WNV sequences generated for 2023 (left) and 2024 (right). The colored icons (orange or red) mark the geographical origins of WNV whole genome sequences from wild and captive birds as indicated by icon shapes (triangle or circle). Grey icons show the origin of WNV RNA positive birds that were not whole genome sequenced. The location of the geodata originates from the German animal notification system (TSN)

Figure 3 illustrates maps of Germany from 2023 and 2024, demonstrating the presence of WNV RNA positive birds sequenced within this work and their distribution. The maps presented herein depict the sequenced samples in different colors, distinguishing between those obtained from captive and wild birds, and their association with the respective cluster or subcluster.

**Fig. 4:**
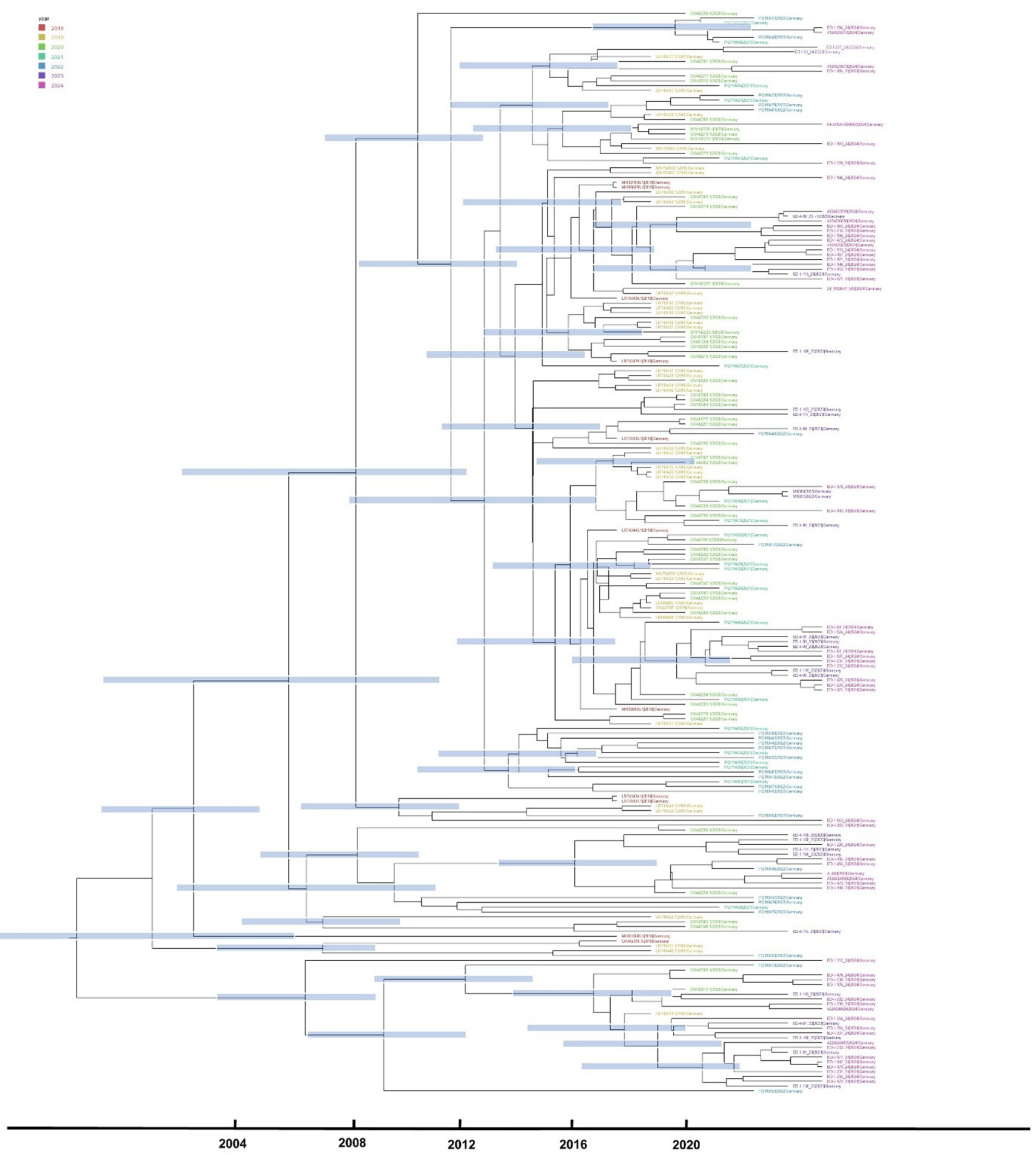
Time scaled molecular clock phylogeny of complete coding sequences exclusively from Germany of WNV lineage 2. Blue bars represent 95% highest posterior density interval for node date estimates. Tip colors indicate year of sampling as described in the legend (upper left corner)

To investigate the temporal origin of WNV-2 circulation in Germany, a time-scaled molecular clock phylogeny was constructed using complete coding sequences (Fig. 4). This analysis focused exclusively on German sequences to examine patterns of virus introduction and local diversification within the country over time. Most WNV-2 sequences from Germany clustered into a single well supported monophyletic group. The estimated tMRCA was dated to approximately mid-2018, with a 95% highest posterior density interval ranging from early 2017 to late 2019. The mean substitution rate for the WNV sequences analyzed was estimated at 8.0 × 10^−4 substitutions per site per year (95% HPD: 5.5 × 10^−4 to 1.1 × 10^−3), This estimate supports the observed temporal signal in the dataset and complements the inferred time to the most recent common ancestor.

**Fig. 5:**
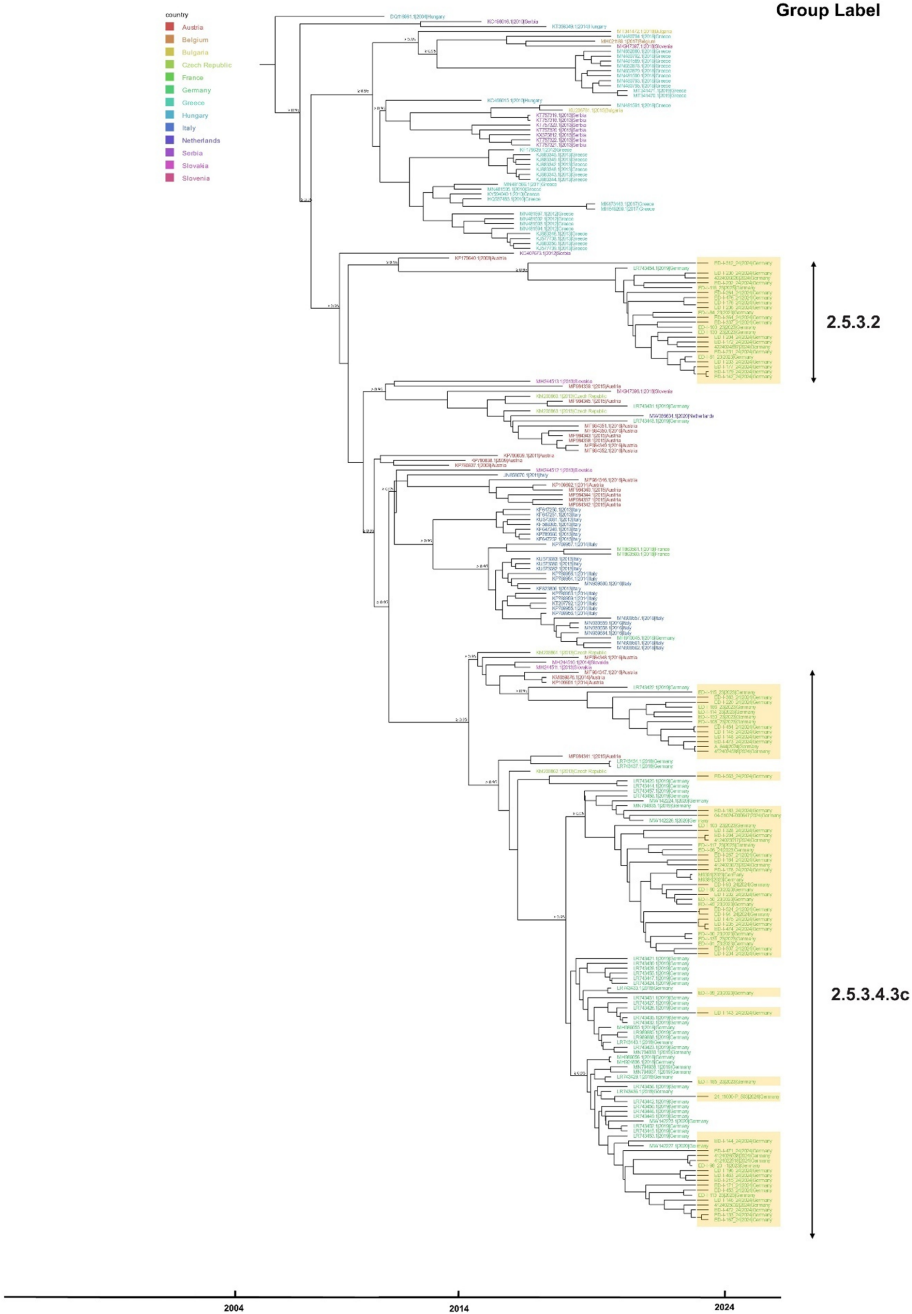
Time scaled phylogenetic tree of complete coding sequences of WNV lineage 2 from Germany and other European countries. Branches with strong support (posterior probability ≥ 0.95) are indicated. Sequences are color-coded by country of origin as described in the legend (upper left corner)

To provide a broader phylogenetic context, we constructed a separate time-scaled phylogeny including WNV-2 sequences from Germany alongside those from other European countries (Fig. 5). Sequences from Germany clustered predominantly within the 2.5.3.4.3c subcluster, which also included closely related sequences from Austria, Czech Republic and Slovakia. Almost all 2.5.3.4.3c sequences, including those from federal states which had not been affected prior to 2024, clustered closely together with sequences from Germany from preceding seasons. Only one sequence from a WNV infected Eurasian Goshawk discovered in Baden-Wurttemberg in November 2024 (ED-I-563/24, Supplementary Table S2) shows greater genetic divergence from other local samples and is genetically closer to a specimen from the Czech Republic collected in 2013.

To gain a clearer understanding of the 2.5.3.2 cluster, we constructed a separate tree focusing exclusively on all available avian sequences from Germany within it (Supplementary Figure SF1). This phylogenetic tree had two main branches. The 23 sequences from 2023 to 2024 can be found in both branches. The first available sequence from 2019 (LR743454) and two sequences found in 2022 (PQ119470 and PQ119453) belong to the larger branch, while two sequences from 2020 (OX442272 and OX442305) cluster in the second main branch. The majority of 2.5.3.2 sequences (2023/2024) originated from known WNV hotspot areas in Germany. Nevertheless, two sequences from Lower Saxony found in 2024 were also part of this cluster and divided between the two aforementioned branches.

## Discussion

Today, WNV is widely present in Europe and strains of WNV-1 and WNV-2 are associated with human disease. WNV-1 was probably introduced from Northern-Western African countries to Italy or France, while WNV-2 was most likely first introduced into Hungary from Southern Africa [35]. Despite the co-circulation of WNV-1 and WNV-2 in Europe, only lineage 2 has been found to circulate in Germany since its first detection in 2018 [6]. Likewise, all avian samples from Germany analyzed in 2023 and 2024 belong to WNV-2. This is consistent with the recent shift in prevalence from WNV-1 to WNV-2 being the predominant lineage in Europe and its emergence into more northern regions, favored by bird migration, which in turn facilitates the transmission process [36]. Thus, WNV-2 remains the dominant strain in Germany and is establishing regionally without any discernible introduction of WNV-1. Furthermore, it has been documented that the vast majority of the infected birds submitted by a contributor in Berlin, who provided numerous samples of Eurasian Goshawks, were juveniles. In this case that means they hatched in the same year, which suggests local virus transmission for these birds. However, further studies are needed to determine the age distribution of positive tested birds and to gain a more profound understanding of how the age of different bird species affects their infection with WNV.

The nomenclature defined by Santos et al. [24] is a good standard for local WNV-2 detailed analysis, which is why it was chosen in this study. Consistent nomenclature over the years is essential for long-term sudies and for comparing the sequences belonging to the clusters shown. The dominant 2.5.3.4.3c subcluster in Germany exhibits a high degree of similarity with sequences previously found in Austria, Czech Republic, and Slovakia. Sequences from the Czech Republic and a human strain from Austria are part of the so-called Central European subclade II, which is included in cluster 2.5.3.4 [6, 24]. As demonstrated by Ziegler et al. [11], the probability of WNV being introduced to Germany from the Czech Republic in 2016 is high [6]. Phylogenetic analyses show that local evolution in southern and central Europe is characterized by the presence of subcluster 2.5.3.4.3a for more than 10 years [24]. The subcluster 2.5.3.4.3c possesses a high degree of genetic similarity thereto. In 2021 and 2022, 95% of the WNV sequences in Germany clustered within the 2.5.3.4.3c subcluster of WNV-2 as shown by Schopf et al. [23]. In both following years, 2023 and 2024, it was again the dominant subcluster. This indicates that the majority of WNV circulation in Germany relies on local transmission rather than continuous external introductions. The phenomenon of few incursory events followed by permanent local establishment has been described as a general characteristic of WNV-2 strains [35].

Even though subcluster 2.5.3.4.3c has always been dominant in Germany, it is not the only detected strain [23, 24]. In our study, this is reflected by a smaller number of sequences belonging to cluster 2.5.3.2 (2023: five sequences, 2024: 18 sequences). Santos et al. [24] and Schopf et al. [23] could not determine whether the five sequences of this cluster detected in Germany between 2019 and 2022 were the result of local circulation or of distinct incursions. In our study, more than 20 generated sequences belonged to this cluster and the corresponding samples were predominantly sourced from birds that are either resident, zoo, or aviary birds. Consequently, it is highly probable that these birds were locally infected with WNV by mosquito bites. This would mean that the 2.5.3.2 cluster is also circulating enzootically in Germany. Further studies will be needed to determine the timepoint of introduction into the country for this cluster. Schopf et al. demonstrated that, for the years 2021 and 2022, WNV RNA positive bird samples were identified predominantly in the eastern and central-eastern regions of Germany [23]. This was also evident in 2023, as almost all WNV RNA positive samples originated from only five federal states (Berlin, Brandenburg, Saxony, Saxony-Anhalt, and Thuringia), the endemic area where WNV has been circulating since its introduction into Germany. Notably, a single positive sample was obtained from Rhineland-Palatinate, a federal state in the southwestern region of Germany. In contrast, in 2024, 20 of the 83 bird samples (~ 24%) that tested WNV RNA positive originated from Baden-Wurttemberg, Bavaria, Hamburg, Hesse, Lower Saxony, Mecklenburg-Western Pomerania, North Rhine-Westphalia, and Schleswig-Holstein, demonstrating the spread of WNV throughout Germany with 13 of 16 federal states being affected. Most sequences originating from newly affected federal states cluster close to those from known hotspot areas. This is true for the 2.5.3.4.3c subcluster as well as the 2.5.3.2 cluster. While the former has been detected in all of the aforementioned federal states in 2024, the latter could only be found in Lower Saxony in addition to the north-eastern hotspots in that year. These phylogenetic findings lend further credence to the hypothesis of silent WNV circulation and local spread within Germany which had been proposed by Schopf et al. [23] on the basis of serological monitoring results. While the former has been detected in all of the aforementioned federal states in 2024, the latter could only be found in Lower Saxony in addition to the north-eastern hotspots in that year. Genetically more distanced sequences, such as the one isolated from a Eurasian Goshawk in Baden-Wurttemberg, demonstrate that viruses do not adhere to national borders and that novel strains can be introduced into local populations alongside stable enzootic circulation. This indicates that the nationwide geographic expansion in 2024 was largely driven by the dominant 2.5.3.4.3c subcluster, while the 2.5.3.2 cluster played a more localized role, restricted to Lower Saxony and the established north-eastern hotspots.

As WNV transmission is seasonally dependent on the vector, and as climate factors play a pivotal role in the number of infections, the reasons for the apparent nationwide emergence of WNV in 2024 may lie within these complex interactions. In 2023 and 2024, respectively, the initial avian cases identified in Germany exhibited temporal proximity (July 8, 2023; July 5, 2024), while the final records differed by more than one month. In 2023, the final case was recorded in Berlin on October 6, whereas in 2024, when a substantially higher number of WNV positive birds were identified, the final case was a Eurasian Goshawk from Baden-Wurttemberg on November 21. Meteorological data from the German Weather Service suggest that both years presented favorable conditions for the dispersal of the vectors [35, 37]. In 2023, a temperate onset of winter, prolonged warmth during late summer and elevated precipitation created optimal conditions for *Culex pipiens* complex development, as this species reproduces in stagnant water reservoirs formed by high rainfall [35, 38]. The year 2024 was characterized by exceptionally high temperatures throughout spring and summer. Elevated average temperatures over an extended period can amplify the risk of WNV transmission via mosquito vectors [39]. Additionally, periods of excessive rainfall in 2024 likely promoted breeding site availability for mosquitoes, while high temperatures may have enhanced contact rates between mosquitoes and avian reservoir hosts congregating around water sources [40]. The simultaneous circulation of subcluster 2.5.3.4.3c and cluster 2.5.3.2, coexisting chains of infection, and the aforementioned increased occurrence of vectors facilitate increased WNV transmission and thus bring the relevance of WNV to public health into sharper focus. It is important to consider the influence of vector activity and bird movement on WNV transmission rates, particularly in the context of public health surveillance measures. For instance, the surveillance zones for mosquito breeding sites should be adapted to precipitation conditions, as these influence the habitats of the vectors.

As WNV is an arborvirus that also infects humans as accidental hosts, the monitoring of the bird population in Germany is of great importance for the assessment of the potential health risk to humans. In 2019, the RKI reported the first five cases of autochthonous, mosquito-borne WNV infection in humans in Germany [15]. In subsequent years, there was a continued presence of humans infected with WNV within the country. The geographical areas in which human WNV cases were documented coincided with those in which the FLI reported evidence of infection in birds and horses [14]. In the years 2023 and 2024, which are the focus of this study, the RKI reported seven and 35 human WNV cases in Germany, respectively, which corresponded regionally with the cases observed in birds [41, 42]. It is noteworthy that in 2024, autochthonous human WNV cases were detected for the first time in the northwestern part of Germany, which coincided also with cases in birds [42]. The geographical distribution of WNV RNA detection in avian hosts extends beyond the areas of human WNV detection. This underscores the necessity for systematic bird monitoring, as it facilitates the early detection and prediction of the hypothesized silent spread of WNV, thereby enabling a better risk assessment. The continuous monitoring of WNV in Germany during 2023 and 2024 revealed expansion of the virus across multiple federal states, with predominance of the subcluster 2.5.3.4.3c of WNV-2. The co-circulation of genetically distinct variants suggests ongoing virus evolution and complex transmission dynamics. Climatic factors facilitating vector proliferation appear to have contributed to increased virus activity and geographical spread. Corresponding human infections further highlight the zoonotic risk and underscore the essential role of integrated surveillance systems.

## Conclusions

This study demonstrates the continuous geographic expansion and endemic establishment of WNV in Germany since 2018. It builds on previously published findings and ensures comprehensive WNV monitoring data throughout Germany. Eurasian Goshawks remain the focus of attention for WNV infections in birds. The predominating WNV-2 subcluster 2.5.3.4.3c, which is closely related to sequences from cases in neighboring countries such as Austria, Czech Republic, and Slovakia, was shown to be driving local transmission. The co-circulation of cluster 2.5.3.2 reflects ongoing viral evolution within Germany. Climatic factors, such as elevated temperatures and rainfall patterns, have contributed to increased vector activity and the further spread of WNV. The zoonotic potential of WNV highlights the importance of an integrated One Health surveillance approach. In order to achieve a comprehensive documentation of WNV infections and to facilitate knowledge-based risk assessments for veterinary and public health, it is crucial to ensure the long-term existence and functionality of nationwide surveillance approaches. This includes the genomic characterization of WNV isolates for a detailed real-time insight into the virus dynamics. Given the transboundary nature of viruses, cross-border collaborations are similarly important for the mitigation of the impact of WNV in the future, e.g. by using coordinated vector control and veterinary and public health strategies.

## Supplementary Information

Supplementary Figure SF1: Phylogeny of avian complete coding WNV-2 sequences of the 2.5.3.2 cluster from Germany 2019-2024.

Supplementary Tables S1 and S2: Whole genome sequenced West Nile RNA positive bird samples in 2023 and 2024.

Figure 2: Phylogenetic tree of WNV sequences from Germany inferred by ML analysis.

Supplementary File F1: Breakdown of the sequences in Figure 5, including their country of origin and total numbers.

Figure 4: Time scaled molecular clock phylogeny of complete coding sequences exclusively from Germany of WNV lineage 2.

Figure 5: Time scaled phylogenetic tree of complete coding sequences of WNV lineage 2 from Germany and other European countries.

Supplementary File F2: BEAST Files.

Figure 1: Number of detected autochthonous cases of West Nile virus infection in Germany from 2018 to 2022.

Figure 3: Geographical origin of WNV sequences generated for 2023 and 2024.

Supplementary Figure SF2: Phylogeny of collapsed German sequences.

## Data Availability

All data generated or analyzed during this study are included in this published article and its supplementary information files. Sequences are uploaded and available on Zenodo (DOI : 10.5281/zenodo.16810627 ) .

## Abbreviations

%: Percentage
cDNA: Complementary deoxyribonucleic acid
e.g.: For example (*exempli gratia*)
FLI: Friedrich-Loeffler-Institut
IgG: Immunoglobulin G
IgM: Immunoglobulin M
MCC: Maximum clade credibility
MCMC: Markov chain Monte Carlo
ML: Maximum likelihood
NRL: National Reference Laboratory
ONT: Oxford Nanopore Technologies
RKI: Robert Koch Institute
RNA: Ribonucleic acid
RT-qPCR: Reverse transcription quantitative real-time polymerase chain reaction
tMRCA: Time to the most recent common ancestor
TSN: German animal notification system
WNV: West Nile virus
WNV-1: West Nile virus lineage 1
WNV-2: West Nile virus lineage 2
WGS: Whole genome sequencing
