# Supplementary material for: Circulation dynamics of West Nile virus in Germany, 2023 and 2024
Source: Virol J. 2025 Dec 18;23:58. doi: 10.1186/s12985-025-03043-8 (PMC12955091; doi:10.1186/s12985-025-03043-8)
Supplement: Supplementary file 1 — Supplementary Figure SF1: Phylogeny of avian complete coding WNV-2 sequences of the 2.5.3.2 cluster from Germany 2019-2024. [file 12985_2025_3043_MOESM1_ESM.pdf]

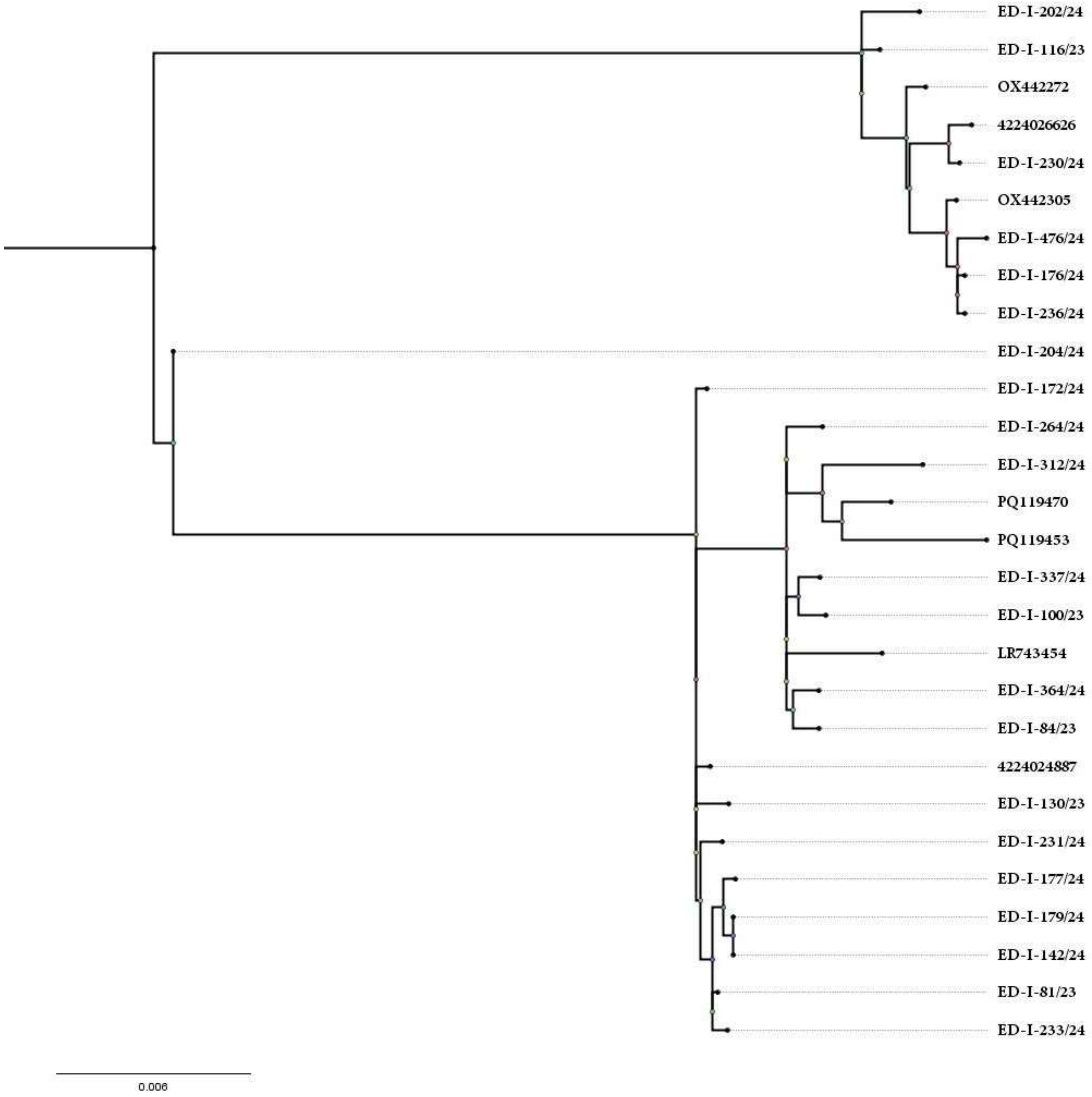

Supplementary Figure SF1: Phylogeny of avian complete coding WNV-2 sequences of the 2.5.3.2 cluster from Germany 2019-2024.
