# Supplementary figures and images for: Circulation dynamics of West Nile virus in Germany, 2023 and 2024
Source: Virol J. 2025 Dec 18;23:58. doi: 10.1186/s12985-025-03043-8 (PMC12955091; doi:10.1186/s12985-025-03043-8)

# Phylogenetic analysis of WNV Lineage 2

Subclade (2.5.3)

Cluster (2)

Subcluster (4.3c)

2023

2024

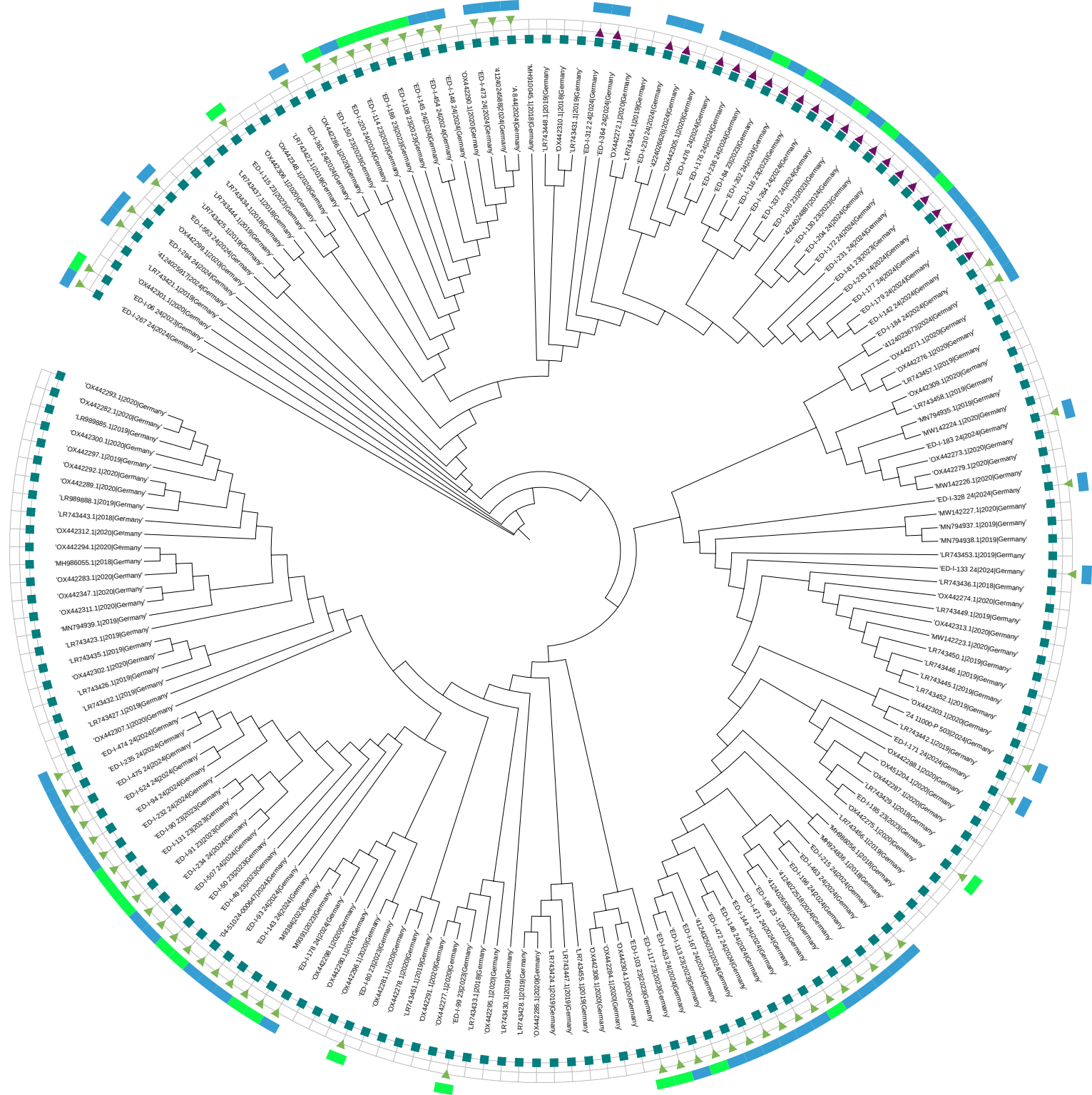

Supplement: Supplementary file 3 — Figure 2: Phylogenetic tree of WNV sequences from Germany inferred by ML analysis. [file 12985_2025_3043_MOESM3_ESM.pdf]

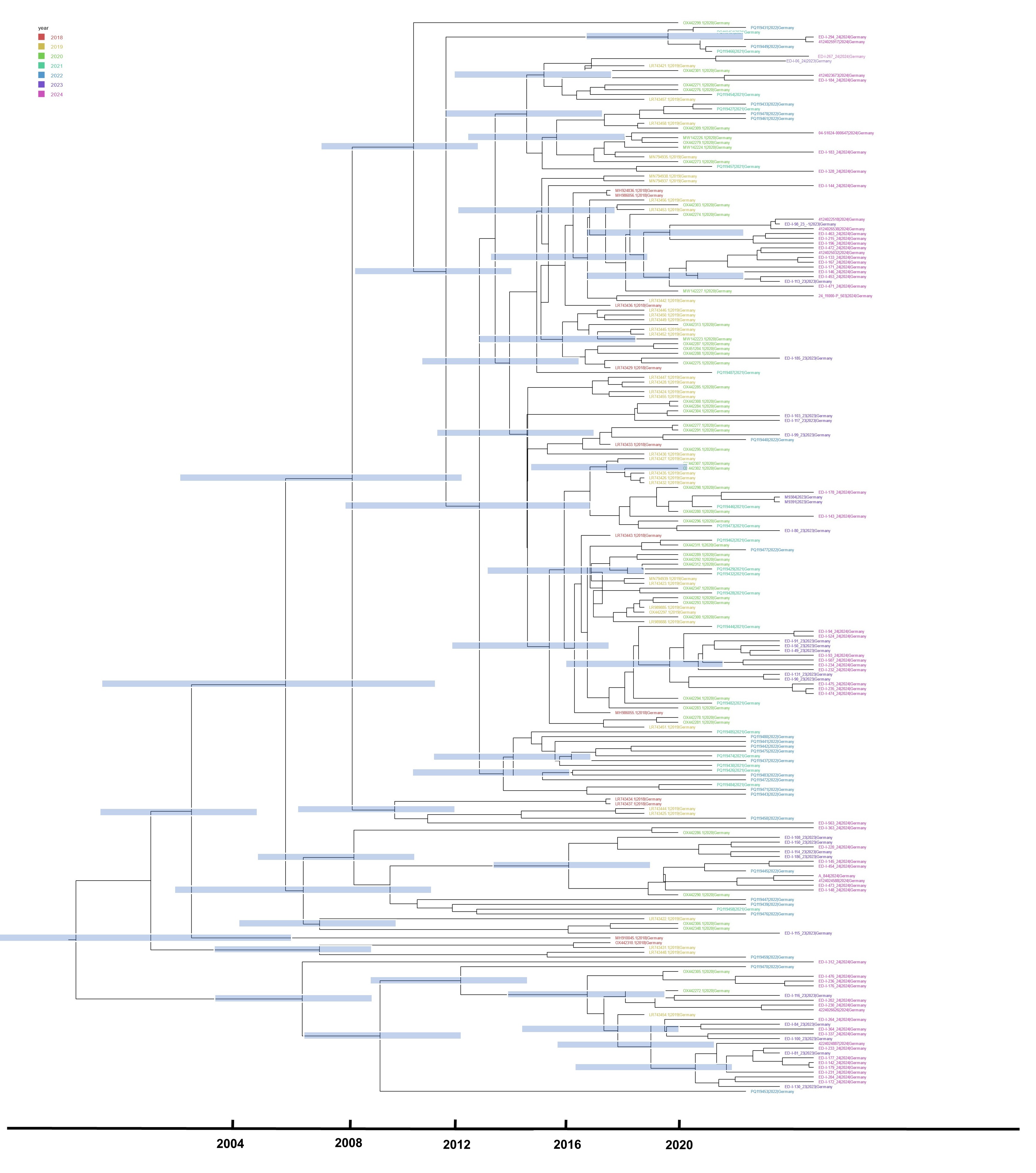

Supplement: Supplementary file 5 — Figure 4: Time scaled molecular clock phylogeny of complete coding sequences exclusively from Germany of WNV lineage 2. [file 12985_2025_3043_MOESM5_ESM.jpg]

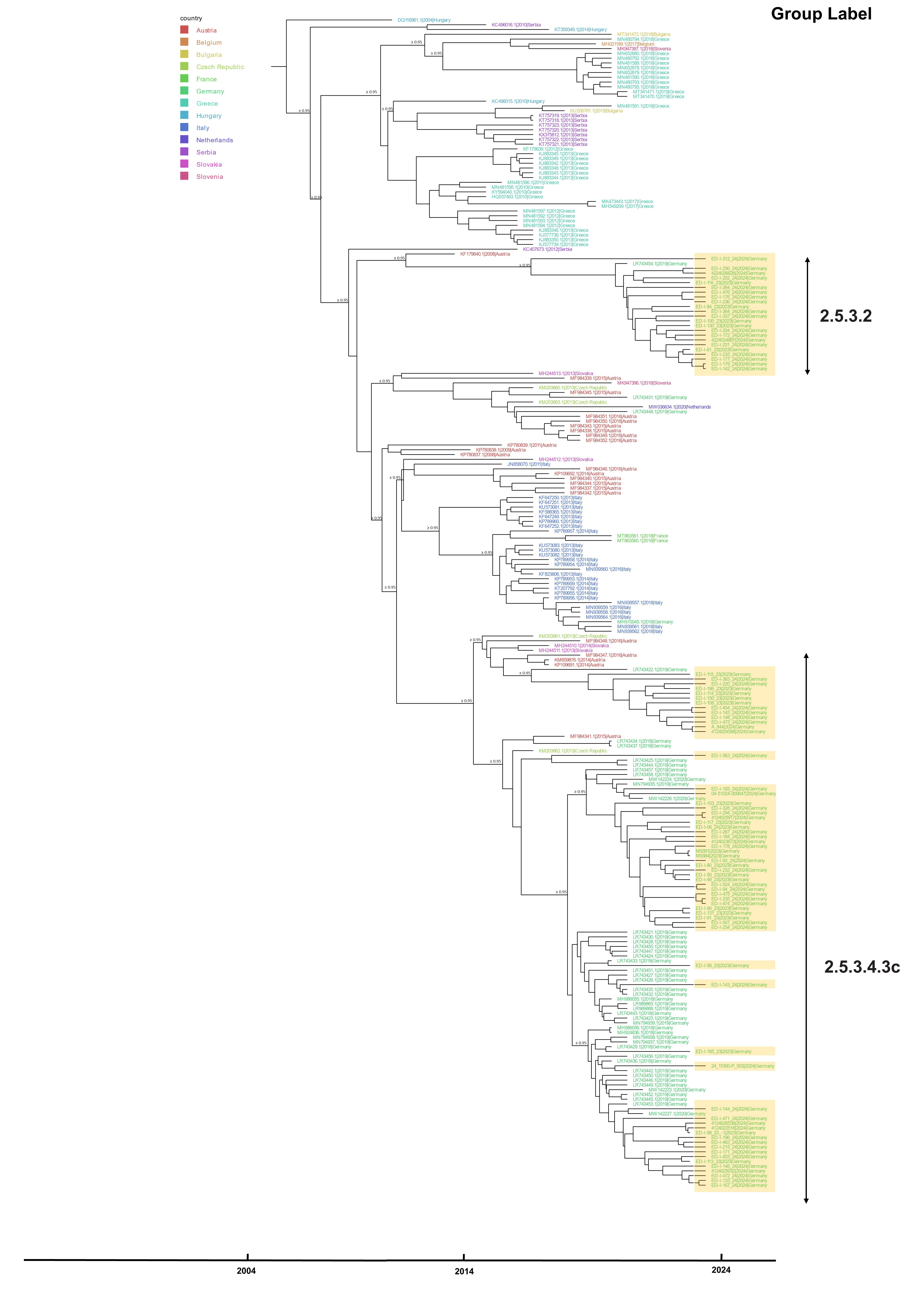

Supplement: Supplementary file 6 — Figure 5: Time scaled phylogenetic tree of complete coding sequences of WNV lineage 2 from Germany and other European countries. [file 12985_2025_3043_MOESM6_ESM.jpg]

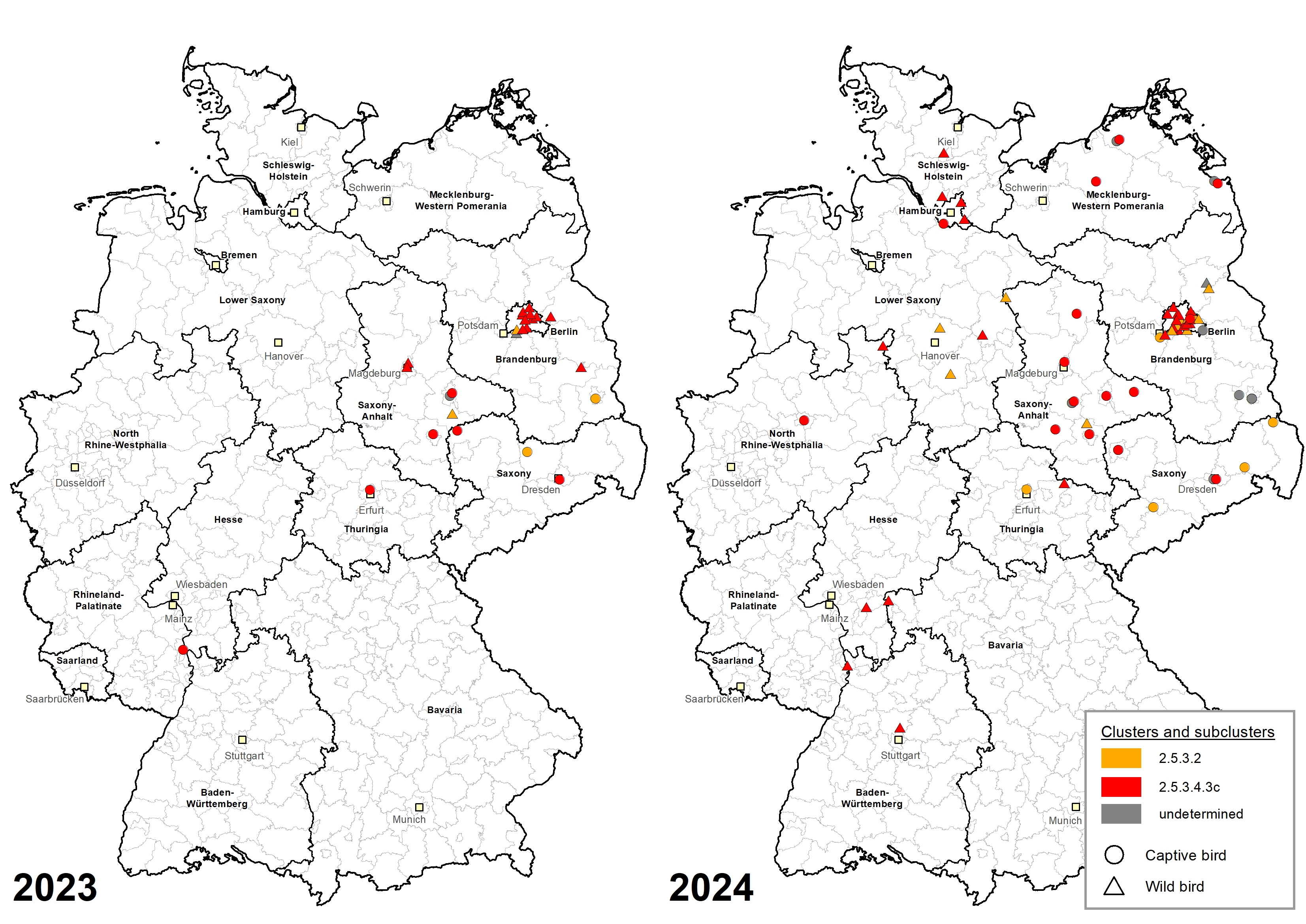

Supplement: Supplementary file 9 — Figure 3: Geographical origin of WNV sequences generated for 2023 and 2024. [file 12985_2025_3043_MOESM9_ESM.jpg]
